# Immune function and blood parasite infections impact stopover ecology in passerine birds

**DOI:** 10.1007/s00442-018-4291-3

**Published:** 2018-11-01

**Authors:** Arne Hegemann, Pablo Alcalde Abril, Rachel Muheim, Sissel Sjöberg, Thomas Alerstam, Jan-Åke Nilsson, Dennis Hasselquist

**Affiliations:** 1 Department of Biology, Lund University, Ecology Building, 223 62 Lund, Sweden; 2 Center for Macroecology, Evolution and Climate, Natural History Museum of Denmark, University of Copenhagen, Universitetsparken 15, 2100 Copenhagen, Denmark

**Keywords:** Avian migration, Eco-immunology, Eco-physiology, Optimal migration

## Abstract

Stopovers play a crucial role for the success of migrating animals and are key to optimal migration theory. Variation in refuelling rates, stopover duration and departure decisions among individuals has been related to several external factors. The physiological mechanisms shaping stopover ecology are, however, less well understood. Here, we explore how immune function and blood parasite infections relate to several aspects of stopover behaviour in autumn migrating short- and long-distance migrating songbirds. We blood sampled individuals of six species and used an automated radio-telemetry system in the stopover area to subsequently quantify stopover duration, ‘bush-level’ activity patterns (~ 0.1–30 m), landscape movements (~ 30–6000 m), departure direction and departure time. We show that complement activity, the acute phase protein haptoglobin and blood parasite infections were related to prolonged stopover duration. Complement activity (i.e., lysis) and total immunoglobulins were negatively correlated with bush-level activity patterns. The differences partly depended on whether birds were long-distance or short-distance migrants. Birds infected with avian malaria-like parasites showed longer landscape movements during the stopover than uninfected individuals, and birds with double blood parasite infections departed more than 2.5 h later after sunset/sunrise suggesting shorter flight bouts. We conclude that variation in baseline immune function and blood parasite infection status affects stopover ecology and helps explain individual variation in stopover behaviour. These differences affect overall migration speed, and thus can have significant impact on migration success and induce carry-over effects on other annual-cycle stages. Immune function and blood parasites should, therefore, be considered as important factors when applying optimal migration theory.

**Electronic supplementary material:**

The online version of this article (10.1007/s00442-018-4291-3) contains supplementary material, which is available to authorized users.

## Introduction

Seasonal avian migration is usually characterised by sequences of movements intermixed with stopovers to refuel and rest. Stopovers are particularly important as theoretical models suggest that 90% of the entire migration time and 67% of all energy consumption is spent during stopovers (Hedenström and Alerstam 1997). Empirical data have supported these assumptions and shown that much more energy is spent during stopovers than during actual flight (Wikelski et al. 2003). The length of stopovers depends on fuelling rates which, together with flight efficiency, determine the overall migration speed (Alerstam and Lindström 1990), and thus the degree of delayed or advanced arrival to the destination (Nilsson et al. 2013), with all its consequences for subsequent annual-cycle stages (Norris and Taylor 2006; Harrison et al. 2011). Hence, stopovers are vitally important for the success of a migratory journey and for individual fitness (Alerstam and Lindström 1990).

Stopovers are primarily needed for refuelling (Lindström 2003) but also to recover from fatigue (Klaassen 1996; Schwilch et al. 2002) or when weather conditions prevent continued migration (Richardson 1978). The length of stopovers and hence the departure decisions are influenced by many factors, including refuelling rate, weather conditions and predation risk (Jenni and Schaub 2003; Schaub et al. 2004; Schmaljohann and Dierschke 2005; Bulyuk and Tsvey 2006). Optimal migration theory predicts that birds maximizing speed of migration should reduce the time spent on stopover sites when fuel deposition rates are high (Lindström and Alerstam 1992; Alerstam and Hedenström 1998) and continue migration as soon as they reach the optimal fuel load (Alerstam and Lindström 1990). Indeed, birds with high body condition usually depart faster from stopover sites compared to lean birds (Biebach et al. 1986; Fusani et al. 2009; Goymann et al. 2010; Lupi et al. 2017). Yet, often much individual variation in stopover duration remains unexplained (Jenni and Schaub 2003; Schmaljohann and Eikenaar 2017).

Proximate mechanisms for departure decisions are linked to hormones, in particular ghrelin and corticosterone, which regulate food intake and body mass, thereby influencing stopover behaviour (Goymann et al. 2017; Eikenaar et al. 2017; Eikenaar 2017). Physiological flexibility of body composition that enables high refuelling rates and efficient flights has also received much attention (reviewed by Piersma and van Gils 2011). Other physiological mechanisms that impact stopover ecology have, however, received little consideration. Recent studies suggest that infected birds exhibit different stopover behaviours (e.g. local movements) and that infections can prolong stopover duration (van Gils et al. 2007; Latorre-Margalef et al. 2009; van Dijk et al. 2015; Risely et al. 2018), suggesting that activating an immune response might play an important role in determining stopover behaviour. The immune system protects the body from diseases and is important for survival (e.g. Roitt et al. 1998; Hegemann et al. 2013b). At the same time, the immune system incurs costs in terms of production, maintenance and activation (Klasing 2004; Hasselquist and Nilsson 2012; Hegemann et al. 2012b). It has, therefore, been hypothesized that immune function is traded-off with other behavioural and physiological processes, in particular behaviours entailing heavy physical workload (Sheldon and Verhulst 1996; Råberg et al. 1998) such as migration (Buehler and Piersma 2008). Indeed, birds modulate and redistribute immune function during migration (Owen and Moore 2008; Buehler et al. 2010; Eikenaar and Hegemann 2016), which can either lead to increased infection risk or increased investment into immune function (Buehler and Piersma 2008). Taken together, trade-offs between immune function and refuelling rate, and hence stopover behaviour, can be expected (Klaassen et al. 2012). Yet, studies investigating if immune function is related to stopover ecology in migrating songbirds, which constitute the majority of avian migrants, are missing.

Departure decisions from a stopover site include not only the decision to leave or stay, but also departure direction and time. Even though birds are expected to migrate in the direction of the destination, reverse migration, i.e. flights in the opposite direction, are commonly observed (Alerstam 1990). Such flights have been linked to body condition, with lean birds having a higher propensity than fat birds to make reverse flights, probably in search for appropriate refuelling sites (Lindström and Alerstam 1986; Deutschlander and Muheim 2009; Nilsson and Sjöberg 2016). Such flights have also been called “landscape movements” and have been classified as part of stopover behaviour (Taylor et al. 2011). Departure time, relative to sunset in nocturnal migrants and sunrise in diurnal migrants, is presumed to be linked to fuel rates and the distance birds can/want to fly. The longer the next flight, the sooner after sunset/sunrise the birds are expected to depart (Schmaljohann and Eikenaar 2017). If trade-offs between immune function and refuelling exist, one might hypothesise that also landscape movements and departure time are linked to immune function, but this has not been examined so far.

Reduction in investment in immune function increases the risk of infections (Roitt et al. 1998). Infections are known to hamper migration performance in a variety of taxa, including mammals (Mysterud et al. 2016), insects (Bradley and Altizer 2005), fish (Sjöberg et al. 2009) and birds (Risely et al. 2018) and we recently demonstrated that an energetically costly mimicked bacterial infection increases stopover duration in songbirds (Hegemann et al. 2018). However, there is no study investigating to what extent mild and/or chronic parasite infections impact stopover ecology in songbirds, which constitute the vast majority of avian migrants. Typical mild infections in migrating songbirds are blood parasite infections (Bensch et al. 2009). Even though a recent study suggests that infections with blood parasites do not impact the aerobic performance of birds (Hahn et al. 2018), no study has yet investigated if infection with blood parasites impacts key aspects of stopover ecology.

Here, we use the increasing possibilities to link physiology to fine-scale movement patterns and stopover behaviour, and present evidence on how immune function and blood parasite infections (i.e., a measure of chronic mild infection) are related to stopover ecology in songbirds. In particular, we tested the effect of baseline immune function and blood parasite infections on stopover duration, bush-level activity patterns, landscape movements, departure direction and departure time. Using automated radio-telemetry, we quantified stopover behaviours and related those behaviours to three measures of baseline innate immune function, one measure of baseline acquired immune function, and to infection with avian malaria parasites. We predicted that parameters of immune function that have been linked to infections [i.e., increased acute phase proteins and complement activity (Buehler et al. 2009; Hegemann et al. 2013a)] as well as blood parasite infections will increase stopover duration while decreasing activity patterns and local movements. Immune parameters that reflect more long-term investment in immune function [i.e., natural antibodies and immunoglobulins (Hasselquist et al. 2001; Versteegh et al. 2014)] are predicted not to be so closely related to parameters of stopover ecology. We also predicted that birds with symptoms of (mild) diseases (e.g. increased acute phase proteins, see above) and/or active parasitemia will depart later in relation to sunset in nocturnal migrants (and sunrise in diurnal migrants) restricting them to migrate in shorter bouts. We included three species of long-distance migrants and three species of short-distance migrants in our study, because these two groups of migrants may face different trade-offs. In particular long-distance migrants have been hypothesised to have reduced immune function due to trade-offs related to resource-demanding barrier crossings (Klaassen et al. 2012).

## Materials and methods

We studied six passerine species during autumn migration 2014 at Falsterbo Peninsula, a stopover site in southwestern Sweden (55.383°N, 12.816°E). Birds were caught as part of the standardised ringing scheme at Falsterbo Bird Observatory (Karlsson and Bentz 2004). We studied ten individuals of each of three long-distance migrants (wintering in sub-Saharan Africa (Cramp 1988)), Tree Pipit (*Ambus trivialis* captured during 29/8–10/9), Willow Warbler (*Phylloscopus trochilus, n *= 11, 1/9–21/9) and Common Redstart (*Phoenicurus phoenicurus,* 2/9–12/9) as well as three short-distance migrants [wintering in Europe (Cramp 1988)], Dunnock (*Prunella modularis,* 12/9–29/9), European Robin (*Erithacus rubecula,* 26/9–11/10) and Song Thrush (*Turdus philomelos,* 4/10–14/10). All birds were captured around the peak migration of each species (i.e., close to the median capture date of all individuals during the standardised ringing scheme), thereby avoiding very early or late migrating individuals (see Supplementary material for capture dates of all birds ringed during the standardised ringing scheme), and in a limited time period to minimise long-term variation in environmental conditions (e.g. food supply, pathogen pressure). All individuals were caught between sunrise (29 August: 5:09; 14 October 6:37) and 10:00 in the morning, and were hatch-year birds. To reduce variation, we made every attempt to avoid very lean or very fat birds and 82% of the 61 birds in total had a fat score of 2–3 according to the scale by (Pettersson and Hasselquist 1985). Our data set included no bird without fat and only one bird (a Song Thrush) with fat score 1. Seven birds had a fat score of 4 (three Robins, three Willow Warblers, one Redstart) and four birds a fat score of 5 (one each of Song Thrush, Redstart, Tree Pipit and Willow Warblers). We had no birds with fat scores higher than 5 in our dataset.

### Blood sampling

Birds were first measured and ringed by staff from the bird observatory. Afterwards, we collected blood samples (~ 100 µl) by puncturing the brachial vein with a sterile needle. Blood samples were kept on ice in an Eppendorf tube until centrifuging for 10 min at 7000 rpm later the same day. Samples were stored at − 50° until subsequent laboratory analysis.

### Radio-telemetry

We used an automated radio-telemetry system at Falsterbo peninsula to determine stopover duration and stopover behaviour, and coded radio-tags were attached to all birds by gluing them to the back of the birds after cutting some feathers on the bird’s back (Sjöberg et al. 2017). The five smaller species were tagged with NTQBW-2 Coded Tags (LOTEK, weight 0.3 g). Song Thrushes were equipped with MST-720-T transmitters (BIOTRACK, weight 1.4 g). The weight of the transmitters never exceeded 4.2% of the birds’ body mass. The automated radio-telemetry system at Falsterbo peninsula (ca. 6 × 6 km) consisting of three receiver stations (SRX600; Lotek Wireless, Newmarket, ON, Canada; one at the capture site and in 2.85 and 5.55 km distance) with 4–5 antennas each (in total 13 antennas) allowed us to estimate stopover duration, departure time and vanishing bearing (hereafter departure direction). We used the stable individual burst rates of the transmitters (2.9–3.1 s) to filter the data by burst rate (Sjöberg et al. 2015). Constant signals over a long period were assumed to be dead birds or transmitters fallen off, and these birds were excluded from the analyses (*n* = 8: 3 Dunnocks, 2 Common Redstarts, 1 European Robin and 2 Willow Warblers). Stopover duration was calculated as number of days between release time and last recorded signal. Using all signals during the last 10 min from the receiver station that was last in contact with a departing bird, we calculated departure direction (vanishing bearing) as a circular mean (Batschelet 1981), weighing each signal by signal strength (Sjöberg and Nilsson 2015). The departure directions were grouped in “forward” (i.e. 135°–315°, SE–NW) or “reverse” (i.e., 315°–135°, NW–SE) migration. We calculated departure time relative to the start of the night (for nocturnal migrants) or start of the day (for diurnal migrants) as a linear variable. As start of the night and start of the day, we subtracted 60 min from sunrise and sunset, respectively, rather than using sunrise and sunset directly, because migrants can depart before sunset or sunrise (Alerstam and Ulfstrand 1975; Åkesson et al. 1996). We used the difference between the last signal of a bird and the start of the night (for nocturnal migrants) or start of the day (for diurnal migrants), as departure time. For details on the telemetry system set up, transmitters and monitoring regime, see (Sjöberg and Nilsson 2015; Sjöberg et al. 2015).

The telemetry system further allowed us to calculate one activity measure and one measure of local movements, which relates to “landscape movements” (Taylor et al. 2011). To quantify small-scale activity (i.e., activity on the “bush-level”), we calculated the variance of the signal strength for each of the 13 antennas. If a bird sits still (e.g., when sleeping) the variance is small, if it moves a lot on the small scale (probably mainly in the range 0.1–30 m and hence indicative of foraging movements) the variance is high. To quantify landscape movements, we calculated the number of antennas that picked up the signal of a bird over a specific sampling period. This measure gives an indication of the local movements of a bird (in the range 30–6000 m). In this measure, the further a bird moves away from the site of capture and the more it moves around the peninsula, the more antennas will pick up the signal. We calculated the activity pattern and the landscape movements for the first 6 h after release, i.e. during daylight hours for all birds but included only those birds that stayed at least six hours to exclude activity related to departure flights. We did not calculate those values for the night as we did not expect birds to move at night, except for departure flights.

### Immune assays

To quantify each bird’s baseline immune function, we measured three parameters of innate immune function and one related to the constitutive part of acquired immune function. Innate immune function is an important first line of defence (Janeway et al. 2005), is related to antigen exposure (Horrocks et al. 2012, 2015) and responds to environmental conditions and immune challenges (Hegemann et al. 2012a, 2013a). Acquired immune function reflects the investment into immune function over longer time scales and hence is related to phenotypic quality (Hasselquist et al. 2001). Specifically, we used a hemolysis-hemagglutination assay to quantify titers of 1) lytic enzymes of the complement system and 2) non-specific natural antibodies (mainly IgM) from preserved plasma samples (Matson et al. 2005). Although high baseline values of lysis titers are thought to be beneficial in terms of general immune defense, lysis titers increase following an immune challenge (Hegemann et al. 2013a). Agglutination titers vary between annual-cycle stages (Hegemann et al. 2012a), but they are more genetically controlled than other immune parameters (Versteegh et al. 2014) and are usually unaffected by acute sickness responses (Matson et al. 2005; Hegemann et al. 2013a). Scans of individual samples were randomized among all plates and scored blindly with respect to individual ID and migratory strategy (by AH). We used a commercially available colorimetric assay kit (TP801; Tri-Delta Diagnostics, Maynooth, County Kildare, Ireland) to quantify 3) haptoglobin concentration in plasma samples (Hegemann et al. 2012a; Matson et al. 2012). Haptoglobin is an acute phase protein that is normally present at low concentrations in plasma but is released from the liver during a pathogenic challenge (Thomas 2000; Cray et al. 2009; Matson et al. 2012). Finally, we used an enzyme-linked immunosorbent assay (ELISA) to quantify 4) the total level of antibodies (immunoglobulins IgY in the circulation) (Sköld-Chiriac et al. 2014; Hegemann et al. 2017).

### Molecular analyses of blood parasites

DNA from preserved blood was extracted using standard phenol/chloroform methods (Sambrook et al. 2002) and subsequently diluted to 25 ng µl^−1^. To determine infection status with the genera *Haemoproteus*/*Plasmodium* and *Leucocytozoon* a nested PCR amplifying a partial segment of the cytochrome *b* gene and using both Haem-F/Haem-R2 and Haem-FL/Haem-R2L primer pairs was applied (Hellgren et al. 2004). We first grouped the data into birds having no infection and birds being infected (independently of if having single or double infection). As double infections can have much stronger negative consequences on individuals than single infections (Marzal et al. 2008), we subsequently also grouped birds into three categories: uninfected birds, birds having one infection (*Haemoproteus/Plasmodium* or *Leucocytozoon)*, and birds having double infections (*Haemoproteus/Plasmodium* and *Leucocytozoon)* with blood parasites.

### Statistics

We used linear mixed models analysed with the program R version 3.2.3 (R Development Core Team 2015). Even though measurements of baseline immune function are often unaltered by short-term handling stress (Buehler et al. 2008; but see Gao et al. 2017), we first checked if handling time and immune parameters were correlated in our dataset, but this was not the case for any parameter (always *F* < 1.17, *p* > 0.29). We then tested if fat had an influence on stopover duration. As expected, fat was not correlated with stopover duration in our dataset (interaction fat*migration type *F*_1,45_ = 0.73, *p* = 0.40; fat *F*_1,46_ = 0.43, *p* = 0.52) probably due to the limited variation in fat score in our dataset (see above). Hence, we did not incorporate fat as a covariate in further analyses. There were no correlations among immune parameters in our dataset (always *F* < 2.13, *p* > 0.17). To test the hypotheses that immune function relates to parameters of stopover ecology, we included the four immune parameters each with the two-way-interaction with migration strategy (long- vs. short-distance migration) in one model. To test the hypothesis that blood parasite infections influenced stopover ecology, we built separate models including the infection status and its interaction with migration strategy. We used a generalised linear mixed model with poisson distribution when having the number of antennas as dependent variable, and with binomial distribution when having the departure direction as dependent variable. For all models, we included species as random effect. We always started with the full model and simplified it using backwards elimination based on log likelihood ratio test with *p* < 0.05 as selection criterion until reaching the minimal adequate model. We used the packages “nlme” for mixed models (Pinheiro 2016), “lme4” for generalised mixed models as well as “gplots” (Warnes 2009) and “ggplot2” (Wickham 2009) for the figures. Marginal R squared values from mixed models were extracted using the package “MuMIn” (Barton 2018). Estimates in the text are mean ± SE.

## Results

### Stopover duration

Stopover duration ranged from 1.7 h to 13.5 days. Long-distance migrants stopped on average for 1.2 ± 0.22 days (i.e., departed 28.8 h after capture) and short-distance migrants for 2.3 ± 0.60 days (i.e., departed 55.2 h after capture) which is a non-significant difference (*F*_1,4_ = 0.86, *p* = 0.40; for data on species, see Electronic Supplementary Material, Table ESM 1).

**Fig. 1:**
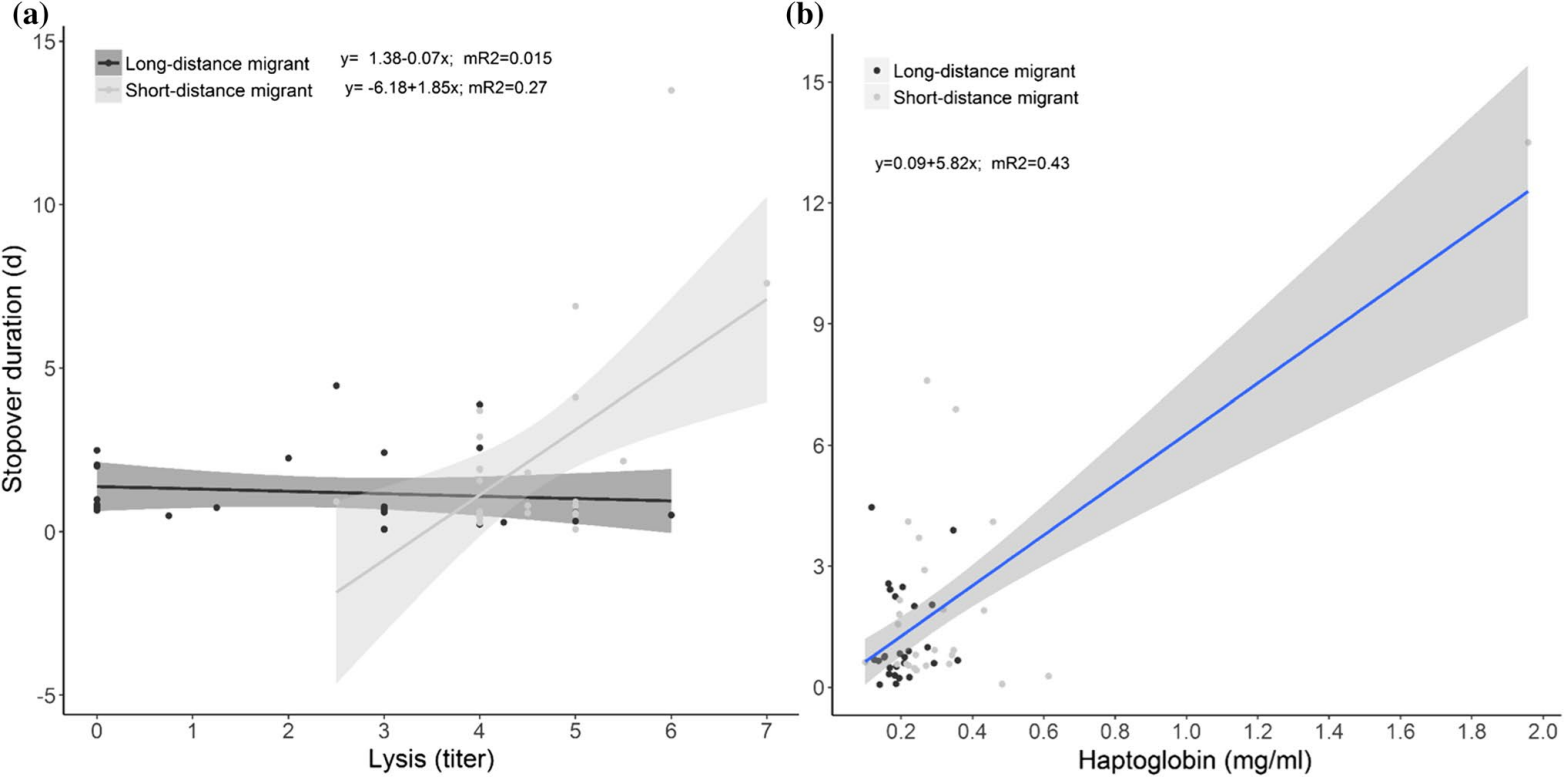
Stopover duration of three species of short-distance and three species of long-distance migratory passerines at Falsterbo peninsula (Sweden) in relation to **a** Lysis (titers) and **b** haptoglobin concentration. The interaction between lysis and migratory strategy was significant. Haptoglobin concentration predicted stopover duration independent of migration strategy, hence we plotted only the line for the pooled data of strategy (See results section for details on statistics). Shading around regression lines represent 95% confidence intervals. mR2 = marginal R squared values from the linear mixed models. See Supplementary material for additional Figures

**Fig. 2:**
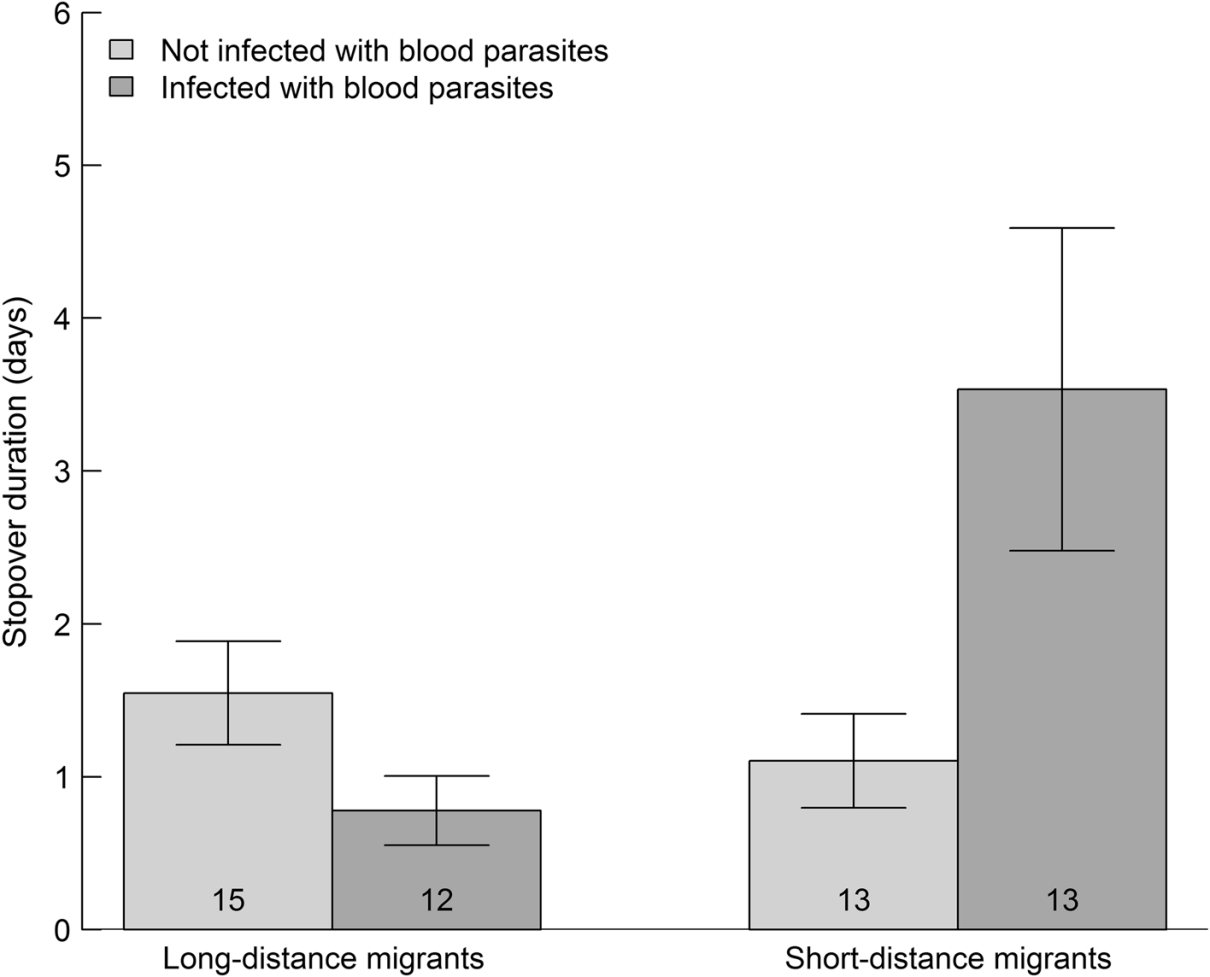
Stopover duration of three species of short-distance and three species of long-distance migratory passerines at Falsterbo peninsula (Sweden) in relation to blood parasite infections. The interaction between infection status (infected/uninfected) and migratory strategy was significant. Numbers in bars represent sample sizes

Stopover duration was related to two immune parameters, lysis (complement activity) and haptoglobin concentration. We found a positive relationship between lysis and stopover duration in short-distance migrants (*F*_1,45_ = 13.4, *p* < 0.001), but not in long-distance migrants (interaction lysis titer*migration type *F*_1,44_ = 5.50, *p* = 0.023; Fig. 1a, Table 1; see ESM for additional Fig). Haptoglobin concentration was positively related to stopover duration in both migration types (*F*_1,44_ = 43.25, *p* < 0.001; Fig. 1b). This relationship depended on one extreme point and was non-significant when this point was discarded (*F*_1,45_ = 0.68, *p* = 0.41). However, we argue that this extreme point is a valid data point and that this relationship is real (see the discussion for the explanation). We found no correlation between stopover duration and agglutination (natural antibodies; *F*_1,43_ = 1.53, *p* = 0.22) or total immunoglobulin levels (IgY, *F*_1,38_ = 0.43, *p* = 0.52). Infections with blood parasites explained variation in stopover duration differently in long- and short-distance migrants (interaction infection*migration type, *F*_1,45_ = 6.95, *p* = 0.011; Fig. 2). In short-distance migrants, infected birds stayed three times as long as uninfected birds, while in long-distance migrants, there was no difference in stopover duration between infected and uninfected birds. Grouping birds into uninfected, infected with single or infected with double blood parasite infections (i.e., having an explanatory variable with three levels rather than two levels) did not change the pattern, and the interaction remained significant (interaction infection*migration type, *F*_2,43_ = 3.88, *p* = 0.028).

**Table 1:** Statistics and coefficients of the linear mixed models of characteristics of stopover behaviour of six species of passerines during autumn migration 2014 in Falsterbo (Sweden)

Y-variable	Model type	X-variable	DF	β	SE	*F*	*P*
Stopover duration	lme	Migration Strategy					
		Lysis (titer)					
		Agg (titer)	1.43			1.53	0.222
		Haptoglobin conc	1.44	5.14	0.92	43.25	**< 0.001**
		Immunoglobulin levels	1.38			0.43	0.516
		Strategy:lysis	1.44	1.04	0.44	5.50	**0.024**
		Strategy:agg	1.37			0.28	0.099
		Strategy:hp	1.35			0.10	0.754
		Strategy:immunoglobulins	1.36			0.22	0.145
Stopover duration	lme	Strategy					
		Blood parasites					
		Blood parasites:Strategy	1.45			6.95	**0.011**
Bush-level activity (variance of signal strength)	lme	Migration Strategy					
		Lysis (titer)	1.35	− 182	96.2	4.76	**0.036**
		Agg (titer)	1.34			0.46	0.502
		Haptoglobin conc	1.33			0.02	0.885
		Immunoglobulin levels					
		Strategy:lysis	1.32			0.11	0.741
		Strategy:agg	1.30			0.01	0.936
		Strategy:hp	1.31			0.02	0.898
		Strategy:immunoglobulins	1.35	− 75.8	32.2	5.51	**0.025**
Bush-level activity (variance of signal strength)	lme	Strategy	1.4			3.75	0.132
		Blood parasites	1.40			1.30	0.260
		Blood parasites:Strategy	1.39			0.10	0.751
Landscape movements (no. of antennas)	Glmer with poisson error structure	Migration Strategy	*			0.42	0.694
		Lysis (titer)				0.23	0.719
		Agg (titer)				0.06	0.935
		Haptoglobin conc				1.06	0.649
		Immunoglobulin levels		0.02	0.01	5.67	**0.017**
		Strategy:lysis				0.43	0.540
		Strategy:agg				0.12	0.696
		Strategy:hp				3.32	0.068
		Strategy:immunoglobulins				0.01	0.899
Landscape movements (no. of antennas)	Glmer with poisson error structure	Strategy	*			1.08	0.299
		Blood parasites				10.22	**0.001**
		Blood parasites:Strategy				0.01	0.969
Departure direction (forward/reverse migration)	Glmer with binomial error structure	Migration Strategy	*			0.57	0.447
		Lysis (titer)				0.01	0.570
		Agg (titer)				2.61	0.100
		Haptoglobin conc				1.06	0.160
		Immunoglobulin levels				0.04	0.544
		Strategy:lysis				0.23	0.567
		Strategy:agg				0.89	0.452
		Strategy:hp				0.79	0.517
		Strategy:immunoglobulins				1.14	0.273
Departure direction (forward/reverse migration)	Glmer with binomial error structure	Strategy	*			0.16	0.684
		Blood parasites				0.13	0.712
		Blood parasites:Strategy				3.22	0.073
Departure time	lme	Migration Strategy	1.4			0.79	0.423
		Lysis (titer)	1.39			0.03	0.863
		Agg (titer)	1.40			0.13	0.717
		Haptoglobin conc	1.42			0.96	0.333
		Immunoglobulin levels	1.41			1.21	0.277
		Strategy:lysis	1.35			0.01	0.964
		Strategy:agg	1.36			0.39	0.535
		Strategy:hp	1.38			1.84	0.183
		Strategy:immunoglobulins	1.37			0.72	0.400
Departure time	lme	Strategy	1.4			2.78	0.171
		Blood parasites	2.45			1.94	0.171
		Blood parasites:Strategy	2.43			0.14	0.714

Species was included as random effect to avoid pseudo replication. Estimates (β) along with their SE (standard error) are only shown for significant terms. When interactions are significant, statistics of main effects cannot be meaningfully interpreted (Looney and Stanley 1989), and, therefore, we do not show these. Final models contain only significant explanatory variables. *p* values < 0.05 are bold

*For generalised linear mixed models with poisson or binomial structure no meaningful degrees of freedom can be produced

### Activity patterns and landscape movement

**Fig. 3:**
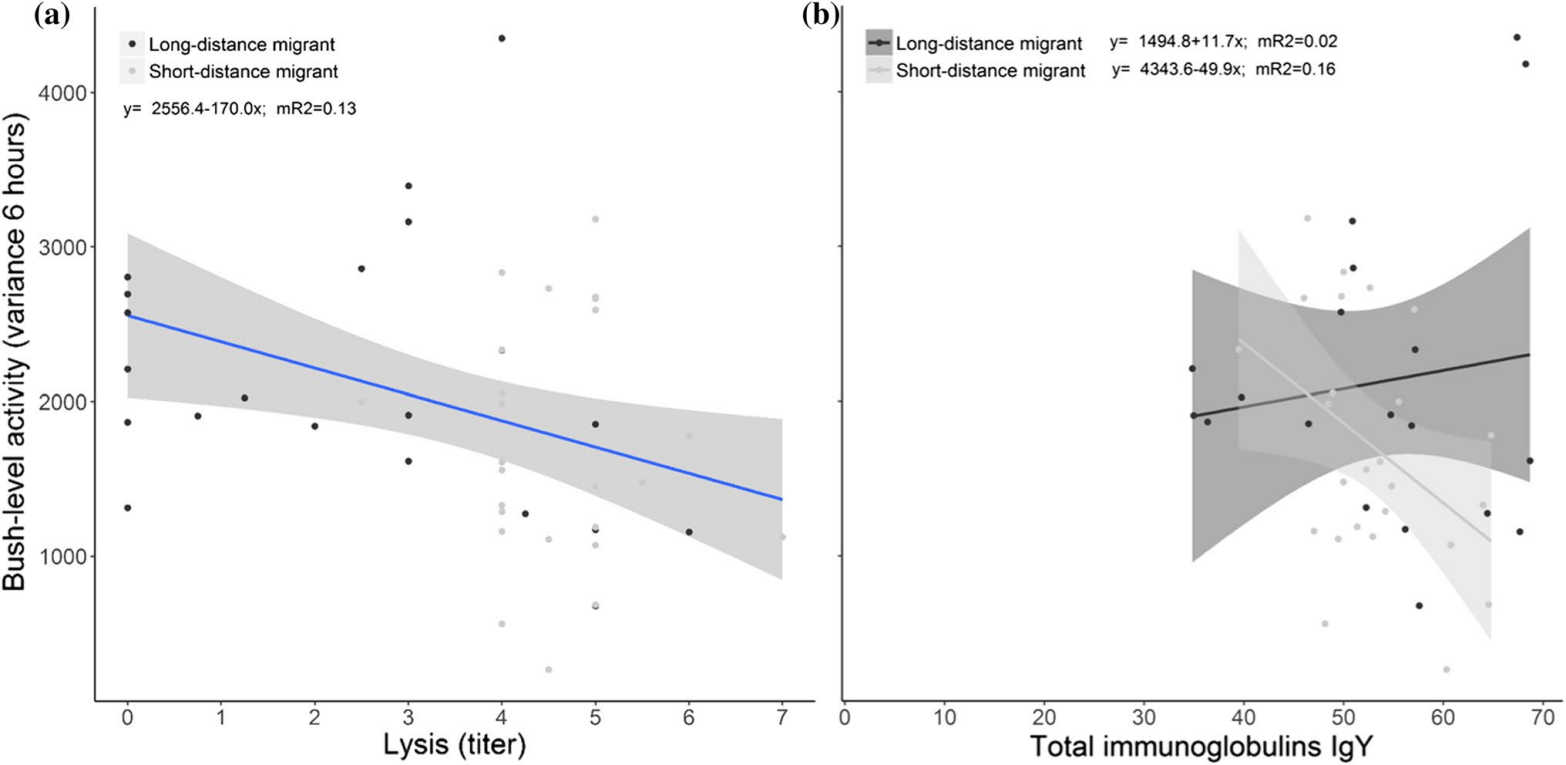
“Bush-level” activity patterns (~ 0.1–30 m) of three species of short-distance and three species of long-distance migratory passerines at Falsterbo peninsula (Sweden) in relation to **a** Lysis (titers) and **b** total immunoglobulins. Bush-level activity patterns were negatively correlated with lysis independently of the migration strategy (long- vs. short-distance migrants), hence we plotted only the line for the pooled data of strategy. Total immunglobulin levels were negatively correlated with bush-level activity patterns in short-distance migrants and unrelated in long-distance migrants (See results section for details on statistics). Shading around regression lines represent 95% confidence intervals. mR2 = marginal R squared values from the linear mixed models

Both bush-level activity patterns and landscape movements were related to immune function. Birds with high lysis showed less bush-level activity than birds with low lysis, independent of the migration strategy (*F*_1,35_ = 4.76, *p* = 0.036, Fig. 3a, Table 1). In short-distance migrants, bush-level activity was negatively correlated with total immunoglobulin, which was not the case in long-distance migrants as evident from a significant interaction (*F*_1,35_ = 5.50, *p* = 0.028, Fig. 3b). We found no relationship between activity and haptoglobin concentrations (*F*_1,33_ = 0.02, *p* = 0.88), agglutination (*F*_1,34_ = 0.46, *p* = 0.50) or infections with blood parasites (*F*_2,40_ = 1.30, *p* = 0.26). Grouping birds into uninfected, infected with single or infected with double blood parasite infections, did not change the pattern (*F*_2,39_ = 1.58, *p* = 0.22).

**Fig. 4:**
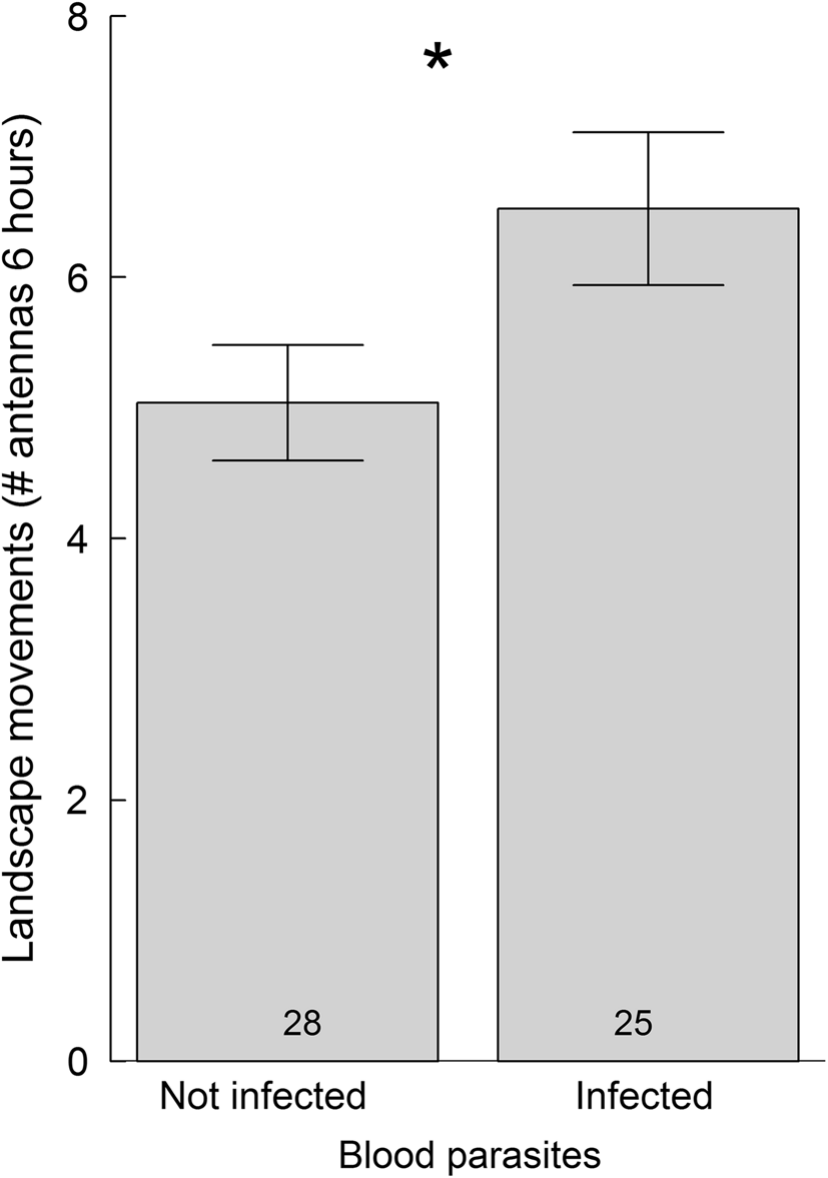
Landscape movements (~ 30–6000 m) during the first 6 h of their stopover (i.e. after capture) of three species of short-distance and three species of long-distance migratory passerines at Falsterbo peninsula (Sweden) in relation to blood parasite infections. Numbers in bars represent sample sizes. The asterisks (*) indicates that groups differ statistically (see results for details)

With respect to landscape movements, birds with high total immunoglobulin levels made more and longer movements on the peninsula than birds with low total immunoglobulin levels (*F* = 5.68, *p* = 0.017, Table 1). Agglutination (*F* = 0.06, *p* = 0.94), lysis (*F* = 0.23, *p* = 0.72) and haptoglobin concentrations (*F* = 1.06, *p* = 0.64) were not related to the extent of local movements on the peninsula. Birds infected with blood parasites showed more landscape movements than uninfected birds (*F* = 10.22, *p* = 0.001, Fig. 4). When grouping birds into uninfected, infected with a single infection and infected with double blood parasite infections (i.e., having an explanatory variable with three levels rather than two levels), there was a trend of increasing landscape movements with increasing infections, i.e. birds with single infections tended to move more than uninfected and birds with double infections tended to move more than birds with single infections (interaction infection*migration type, *F*_2,39_ = 4.59, *p* = 0.10).

### Departure direction and departure time

**Fig. 5:**
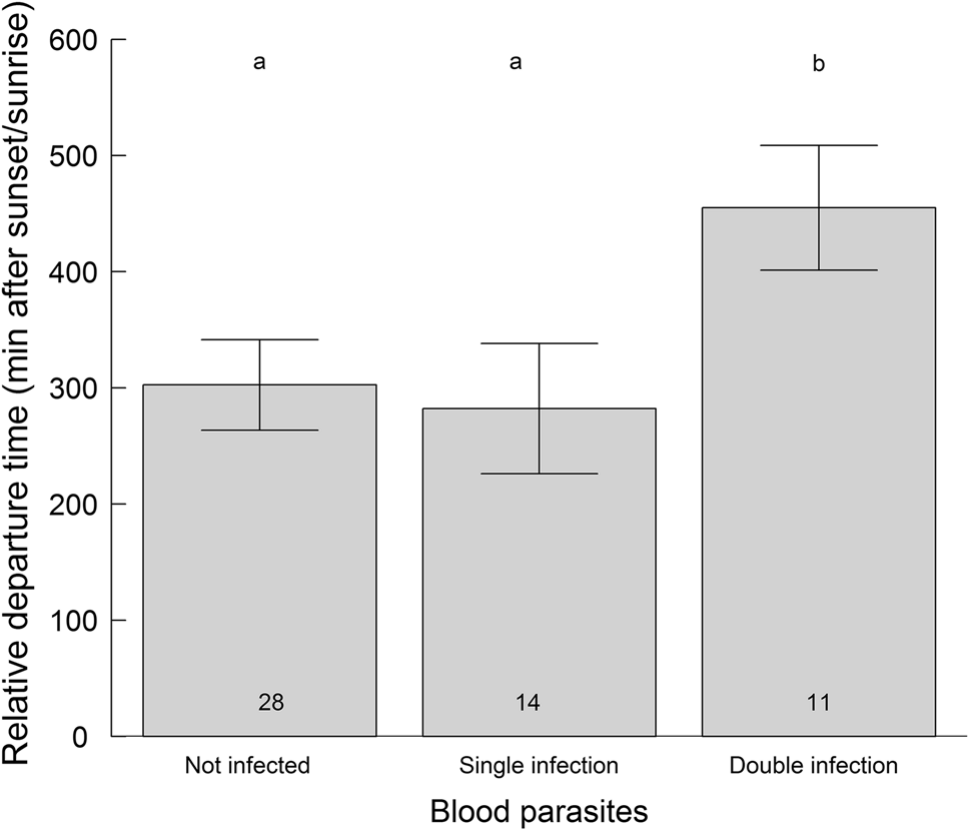
Departure time after sunset/sunrise of three species of short-distance and three species of long-distance migratory passerines at Falsterbo peninsula (Sweden). Birds were either not infected with avian malaria, infected with one (single infection) or with two (double infection) avian malaria lineages. Single infections contain birds only infected with *Haemoproteus/Plasmodium* (*n* = 7) and birds only infected with *Leucocytozoon* (*n* = 7). Letters above bars represent results of Tukey PostHoc test (not infected vs. single infection, *p* = 0.98; not infected vs. double infection, *p* = 0.013, single infection vs. double infection, *p* = 0.016)

Departure direction, classified as forward migration (i.e. 135°–315°) or reversed migration (315°–135°) was not related to any immune parameter (always *F* < 2.6, *p* > 0.10, Table 1) or infection status, either when comparing infected and uninfected (*F* = 0.13, *p* = 0.71) or when splitting infected into single and double infected (*F* = 0.14, *p* = 0.71). Departure time was not related to any immune parameter (always *F* < 1.2, *p* > 0.28) or infection status when comparing infected with uninfected birds (*F*_1,46_ = 1.94, *p* = 0.17). However, birds with double blood parasite infections departed on average more than 2.5 h later after sunset/sunrise than birds without infections or with single infections (*F*_2,45_ = 5.26, *p* = 0.009, Fig. 5).

## Discussion

Our study suggests that several parameters of stopover ecology in autumn migrating songbirds are related to baseline immune function and to blood parasite infections, and that these relationships partly depend on the migration strategy. Thereby, we add a new level of understanding to avian stopover ecology by extending the analysis of physiological and environmental factors to also include several immune-related intrinsic factors previously not measured in relation to stopover ecology and not incorporated into models of migration ecology. On the basis of these data, we present physiological proximate mechanisms (other than hormonal regulations) that explain variation in key parameters of stopover ecology, which are crucial for optimal migration theory (Alerstam and Lindström 1990; Alerstam 2011). That immune function is a potential important determinant of stopover ecology had not been identified in recent conceptual models (Müller et al. 2016; Schmaljohann and Eikenaar 2017) and our findings, therefore, may have important implications for migration research.

Stopover duration, a key parameter of stopover behaviour that reflects refuelling efficiency which is regulated by different environmental and physiological factors (see introduction), was significantly positively related to two parameters of innate immune function and to blood parasite infections. This suggests that immune function is linked to stopover duration in accordance with our predictions. Our findings also refine the general assumption that “body condition” is linked to prolonged stopover duration (Müller et al. 2016; Schmaljohann and Eikenaar 2017). In our study, stopover duration was related to complement activity (lysis), but this relationship was only evident in short-distance migrants. Long-distance migrants had on average much lower lysis than short-distance migrants and lysis was not related to stopover duration in this group of migrants. One possible explanation for this strategy-dependent difference is that long-distance migrants, which are supposedly more time minimisers and under stronger selection pressure to migrate fast than short-distance migrants (Alerstam and Lindström 1990), suppress investment into immune function and instead use resources to keep up migration speed when compared to short-distance migrants. Short-distance migrants had relatively higher lysis, which may suggest that they invest more in immune function during migration which may reduce the probability of disease-related mortality (Hegemann et al. 2015). Moreover, lysis was related to stopover duration in short-distance migrants which may have two, not mutually exclusive explanations. First, maintaining high lysis may be resource-consuming and individuals investing heavily in this parameter of immune function may trade off resource investment in immune function against high fuel deposition rates (Klaassen et al. 2012). Second, very high lysis can also indicate (mild) infections (Hegemann et al. 2013a), and hence birds with the highest lysis might stop over for longer periods due to ongoing mild or chronic infections. The fact that birds with high lysis were less active, as shown by lower bush-level activity patterns, than birds with low lysis supports this latter hypothesis. Haptoglobin concentration, a marker of inflammation, was positively related to stopover duration. Even though this relationship depended on one extreme point, we argue this reflects a true pattern, which may, however, be a more categorical relationship than a continuous relationship. First, in passerine birds, haptoglobin concentrations in the range of 1.0–2.0 mg/ml or even higher regularly occur (Hegemann et al. 2012a; Vermeulen et al. 2016; Ndithia et al. 2017; Fowler and Williams 2017). Second, haptoglobin concentrations increase during inflammation (Thomas 2000; Buehler et al. 2009; Matson et al. 2012; Hegemann et al. 2013a; but see Schultz et al. 2017) and third, a recent study has shown that birds prolong their stopover duration during an experimentally induced inflammatory response (Hegemann et al. 2018). That we had only one bird in our dataset with very high haptoglobin values is not surprising, because this is a marker of an on-going acute inflammation. When catching birds on a stopover site, one would not expect to catch (many) birds that are having an ongoing inflammation, i.e. birds that are actually sick. That this bird was infected with *Haemoproteus/Plasmodium* could indicate that it was in the acute phase of a malaria parasite infection, but we lack data on infection intensity. Likewise, this bird might have been infected with other pathogens we did not measure (e.g. bacterial or virus infections). Taken together, the relationship between haptoglobin concentration and stopover duration might be categorical or bimodal with no relationship among birds with low concentrations and only a strong effect in birds with high concentrations. Further studies will need to investigate these possibilities.

Natural antibodies (agglutination) were not related to any parameter of stopover ecology. This was expected because natural antibodies are known to be more genetically determined than other immune parameters (Versteegh et al. 2014) and do not change during immune challenges (Hegemann et al. 2013a). Likewise as predicted, total immunoglobulin levels in circulation, a marker of the constitutive part of acquired immune function that reflects more long-term investment in immune function, was unrelated to stopover duration. Surprisingly though, total immunoglobulin levels were negatively related to bush-level activity patterns, which are indicative of foraging behaviour. As with lysis and stopover duration, this relationship was only evident in short-distance migrants and absent in long-distance migrants. We currently lack a good explanation for this negative relationship between immunoglobulins and bush-level activity patterns. A possibility may include that high-quality individuals [as indicated by high immunoglobulin levels, which may reflect a long-term investment in immune function rather than current infections (Garvin et al. 2008; Dunn et al. 2010)] are more efficient foragers and hence need less foraging activity. Alternatively, individuals with low immunoglobulin levels may try to find specific nutrients for immunoglobulin production, resulting in higher bush-level activity. Detailed studies and field experiments will be required to test these hypotheses or to reveal alternative explanations. The lack of a relationship in long-distance migrants is in line with the hypothesis that long-distance migrants suppress investment in immune function during migration (Klaassen et al. 2012).

In our dataset, birds with double blood parasite infections (i.e., infected with two avian malaria-like linages) left the stopover site significantly later in relation to sunset/sunrise than uninfected birds or birds just having one infection (one avian malaria lineage), suggesting that double-lineage infected songbirds fly shorter bouts. This is surprising as a recent experimental study suggests that blood parasites do not impair the aerobic capacities of migrating birds (Hahn et al. 2018). Reasons for this discrepancy in results could be that Hahn et al. measured birds in captivity, i.e., under benign conditions, and only with single infections. In our study, later departure, and hence expected shorter flight distance, was only evident in birds with double infections. This was indeed driven by the effect of having a double infection per se, because being infected with only either *Haemoproteus/Plasmodium* or *Leucocytozoon* did not delay departure time. Only birds being infected simultaneously with *Haemoproteus/Plasmodium* and *Leucocytozoon* departed later. This is in line with data showing that double infections have generally a much stronger impact on life-history traits in birds than single infections (Marzal et al. 2008) and that migratory performance decreased with increasing infection intensity (Risely et al. 2018). Since we measured only avian malaria parasite infections but no other type of infections (e.g. viruses, bacteria), we can, however, not exclude that additional co-infections may contribute to the observed effects. In any case, free-flying birds undergoing infections might not only prolong stopover duration (Hegemann et al. 2018, this study), but pathological sickness symptoms may also reduce their flight range. Interestingly, birds infected with blood parasites showed significantly longer landscape movements (i.e., movements away from the catching site) than uninfected birds and there was a trend that this effect increased from single to double infections. This could be a strategy for these birds to avoid the high competition from a large number of birds at the small isolated woodlot at the catching site and move away to find better feeding sites with fewer resting birds further up the Falsterbo peninsula (cf. Lindström and Alerstam 1986). The double infected birds may need more food resources in order to cope with the costs of the parasites or immune activation costs (Hasselquist and Nilsson 2012; Asghar et al. 2015) and may, therefore, reallocate energy from fat deposition to the immune system and hence refuel at lower rates. This may also explain why they depart later and potentially fly only shorter migration bouts. That short-distance migrants in our study had almost a threefold longer stopover duration when being infected with blood parasites could support this hypothesis. These combined effects can potentially explain costs of malaria-like infections in migrants even though the aerobic capacity is unaffected by malaria parasites (Hahn et al. 2018). If birds with double malaria-like infections delay initiation of migratory flights several hours into the night and hence can fly only shorter migration bouts per night, and in addition have similar or even longer stopovers (the latter evident for short-distance migrants in our dataset), this will inevitably have the consequence of decreased migration speed. Hence, this finding will have important implications for optimal migration theory, because birds with disease will fly much shorter distance per night but still have a similar (or even longer) stopover duration, thus decreasing migration speed. The possible implications of this finding becomes particularly evident when considering that double infections occur in many European migrants (Bensch et al. 2009) and once infected, usually remain in a chronic stage for life, i.e. for multiple migrations (Marzal et al. 2008; Asghar et al. 2011; Marzal et al. 2013).

In conclusion, our data suggest that differences in immune function and parasite infections help to explain individual variation in stopover ecology (i.e., stopover behaviour, duration, departure timing). This may either be due to migrants with mild infections having to pursue a strategy of longer stopovers, or reflect strategic decisions of different levels of investment in immune function. In our study, short-distance migrants seem to invest more in baseline immune function during migration than long-distance migrants, resulting in somewhat longer stopovers in the former group. As stopover ecology is crucial for the speed and timing, and hence the success of migration (Alerstam and Lindström 1990; Nilsson et al. 2013), any parameter that affects stopover ecology can ultimately lead to carry-over effects on other annual-cycle stages and individual fitness (Harrison et al. 2011). Although such effects are best documented for spring migration, optimal timing of autumn migration is also important. Food supply usually deteriorates along the migratory route when season advances, especially for (Eurasian) long-distance migrants (Alerstam and Hedenström 1998, Newton 2008). Furthermore, early migrants have competitive advantage in occupying the best territories at stopovers and during winter. This can lead to carry-over effects to the subsequent breeding season (Studds and Marra 2005). Hence, also the timing of autumn migration can have important consequences for other annual-cycle stages and for individual fitness. Our study suggests that immune function might be a physiological mechanism underlying such carry-over effects between autumn migration and subsequent annual-cycle stages. Effects may be even stronger during spring migration. Understanding these mechanisms will be beneficial if we aim to model carry-over effects and their impact on population trends. It can also help us to understand the spread of diseases by migratory birds, because migration success as well as changes in number and length of stopovers of infected birds will be very important parameters when predicting how efficiently diseases are spreading by the huge number of migrating birds that are moving long distances (even between continents) twice a year.

## Electronic supplementary material

Below is the link to the electronic supplementary material.

Supplementary material 1 (DOCX 797 kb)
