# Supplementary material for: Immune function and blood parasite infections impact stopover ecology in passerine birds
Source: Oecologia. 2018 Nov 1;188(4):1011–24. doi: 10.1007/s00442-018-4291-3 (PMC6244813; doi:10.1007/s00442-018-4291-3)
Supplement: Supplementary file 1 — Supplementary material 1 (DOCX 797 kb) [file 442_2018_4291_MOESM1_ESM.docx]

**Supplementary material**

To the manuscript “Immune function and blood parasite infections impact stopover ecology in passerine birds” by Arne Hegemann, Pablo Alcalde Abril, Rachel Muheim, Sissel Sjöberg, Thomas Alerstam, Jan-Åke Nilsson & Dennis Hasselquist. Oecologia.

Additional Figures:


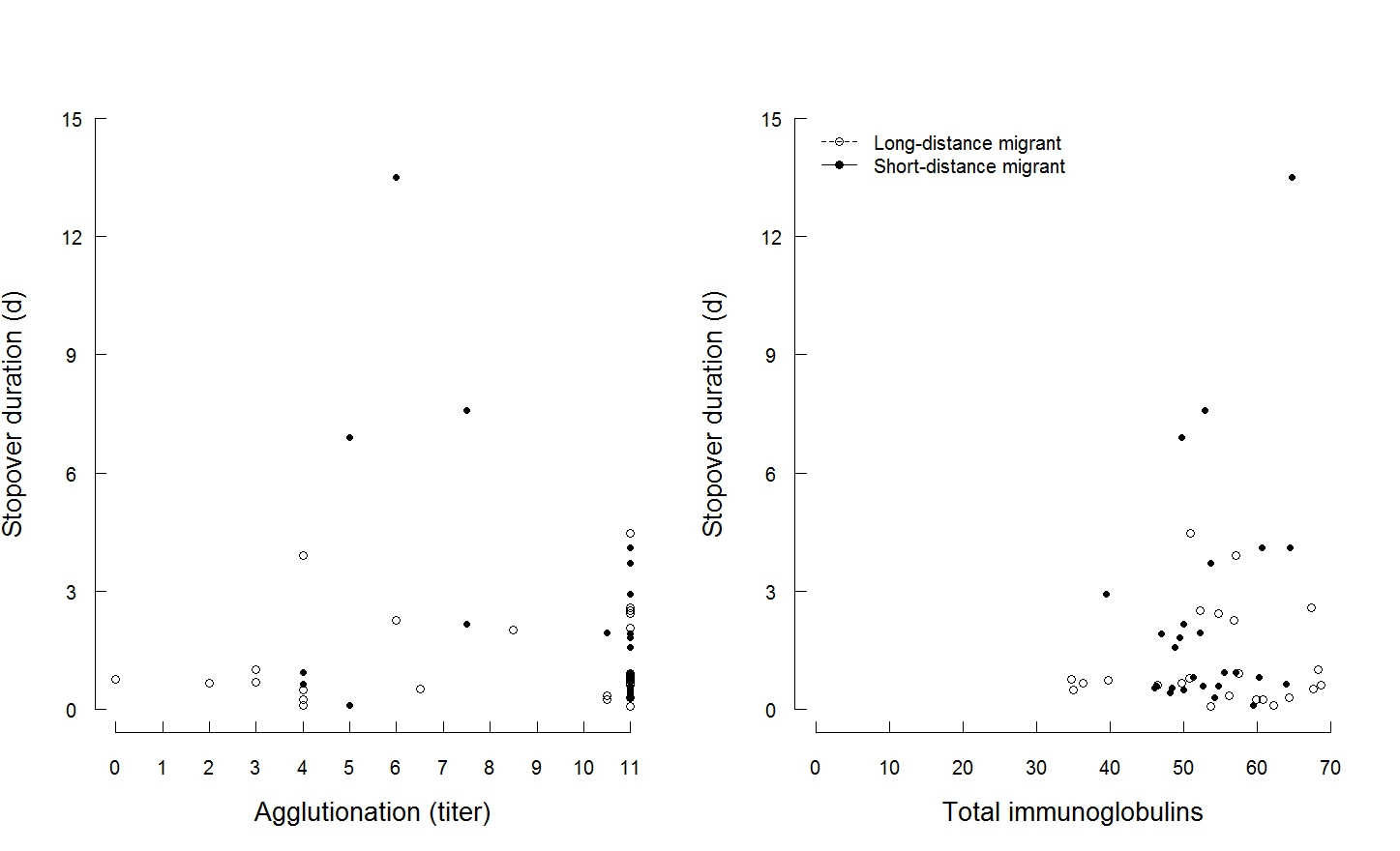


Figure S1: Stopover duration of three species of short-distance and three species of long-distance migratory passerines at Falsterbo peninsula (Sweden) in relation to A) Agglutination (titers) and B) Total Immunoglobulins.


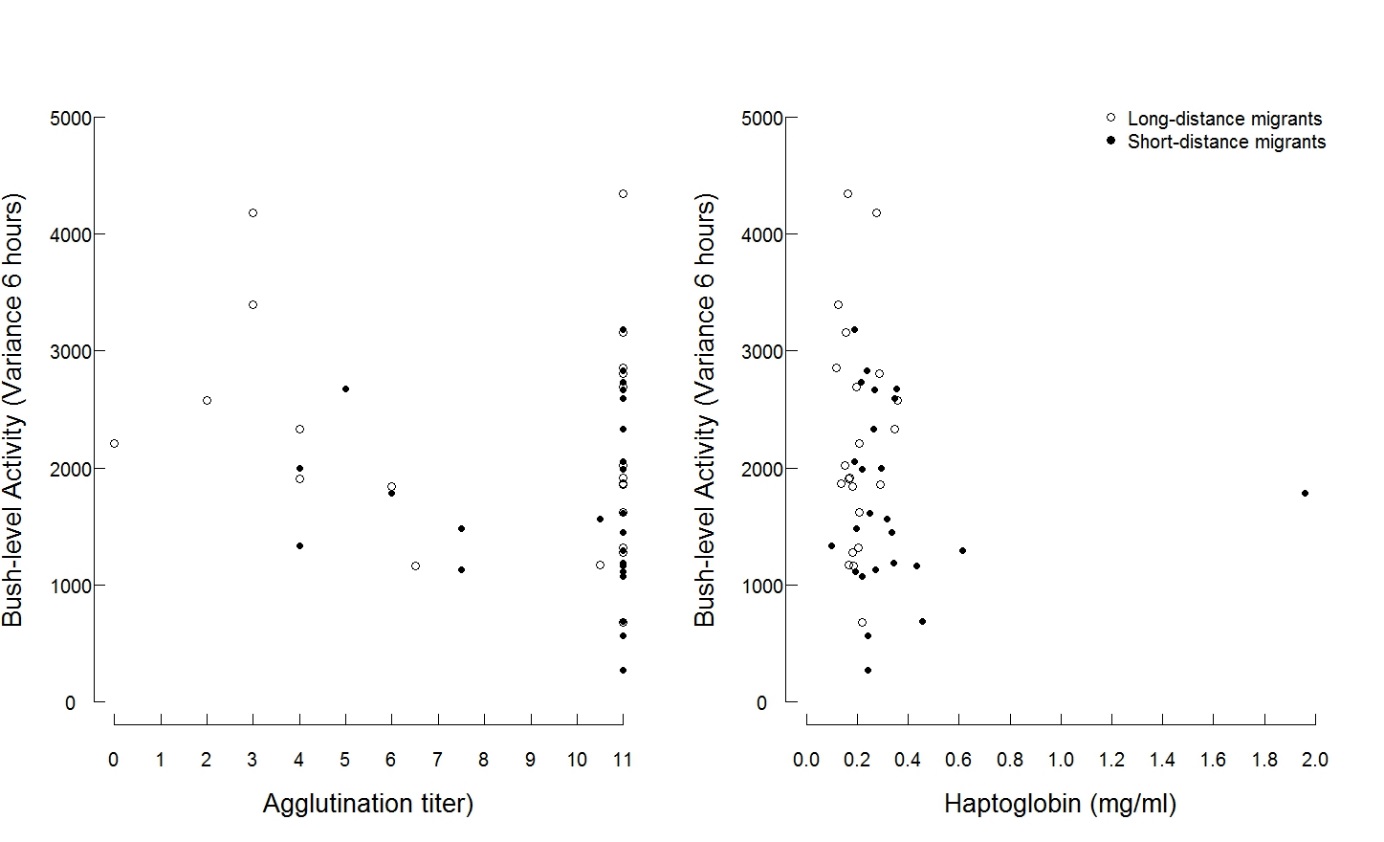
Figure S2: “Bush-level” activity patterns (~0.1-30 m) of three species of short-distance and three species of long-distance migratory passerines at Falsterbo peninsula (Sweden) in relation to A) Agglutination (titers) and B) Haptoglobin concentration.


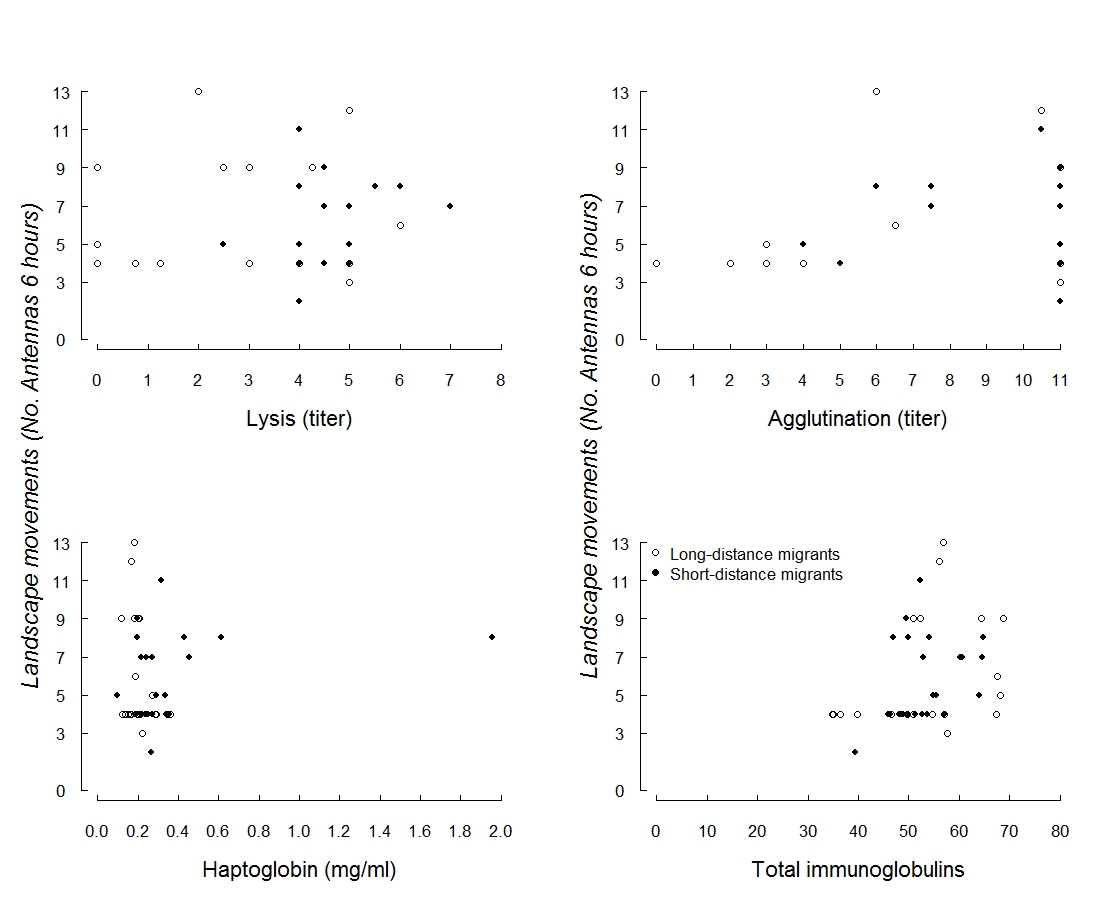


Figure S3: Landscape movements (~30-6000 m) during the first 6 hours of their stopover (i.e. after capture) of three species of short-distance and three species of long-distance migratory passerines at Falsterbo peninsula (Sweden) in relation to immune parameters.


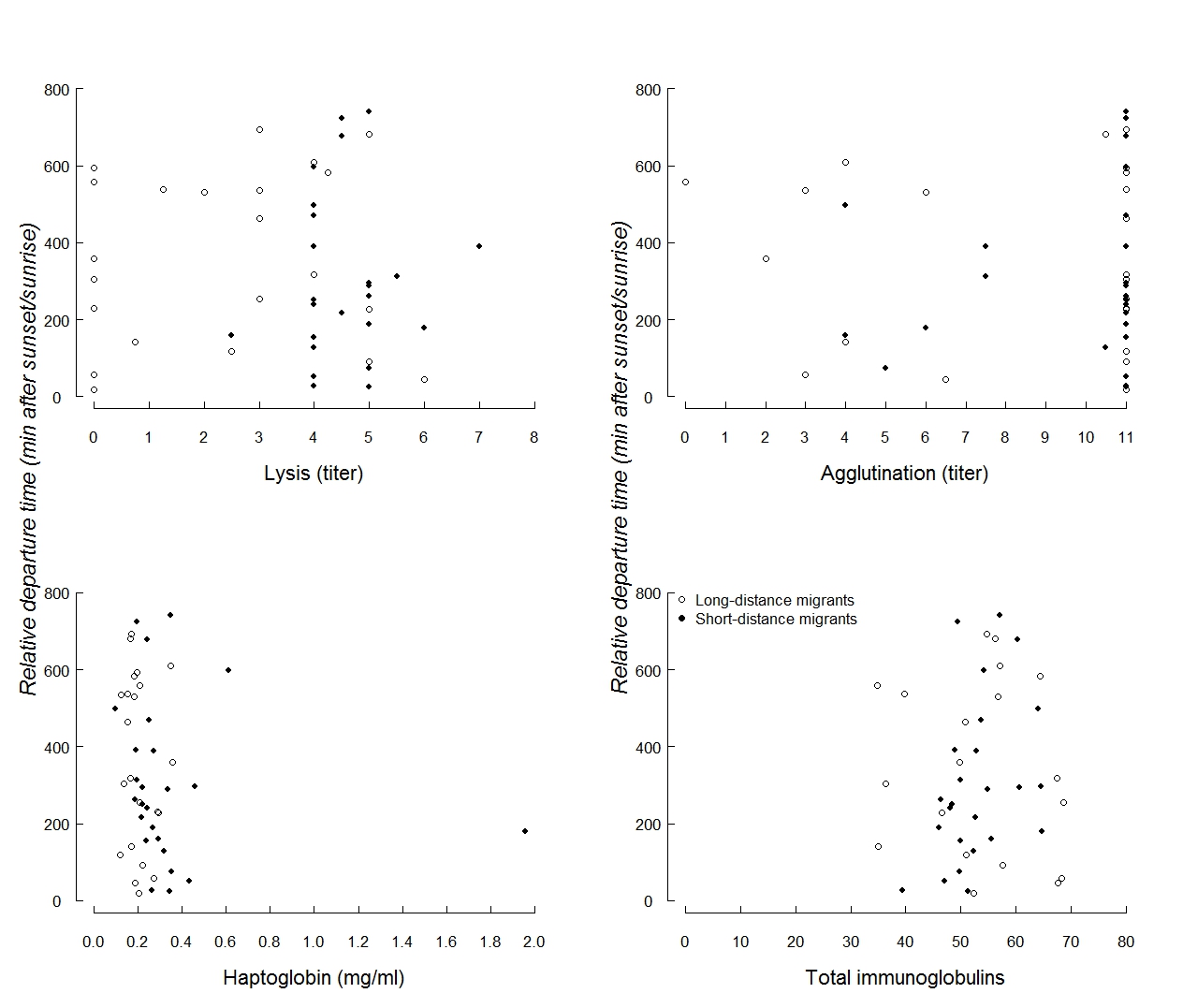


Figure S4: Departure time after sunset/sunrise of three species of short-distance and three species of long-distance migratory passerines at Falsterbo peninsula (Sweden) in relation to immune function. Please note that the blood samples from which the parameters of immune function were quantified were taken on capture which can have been several days before departure.


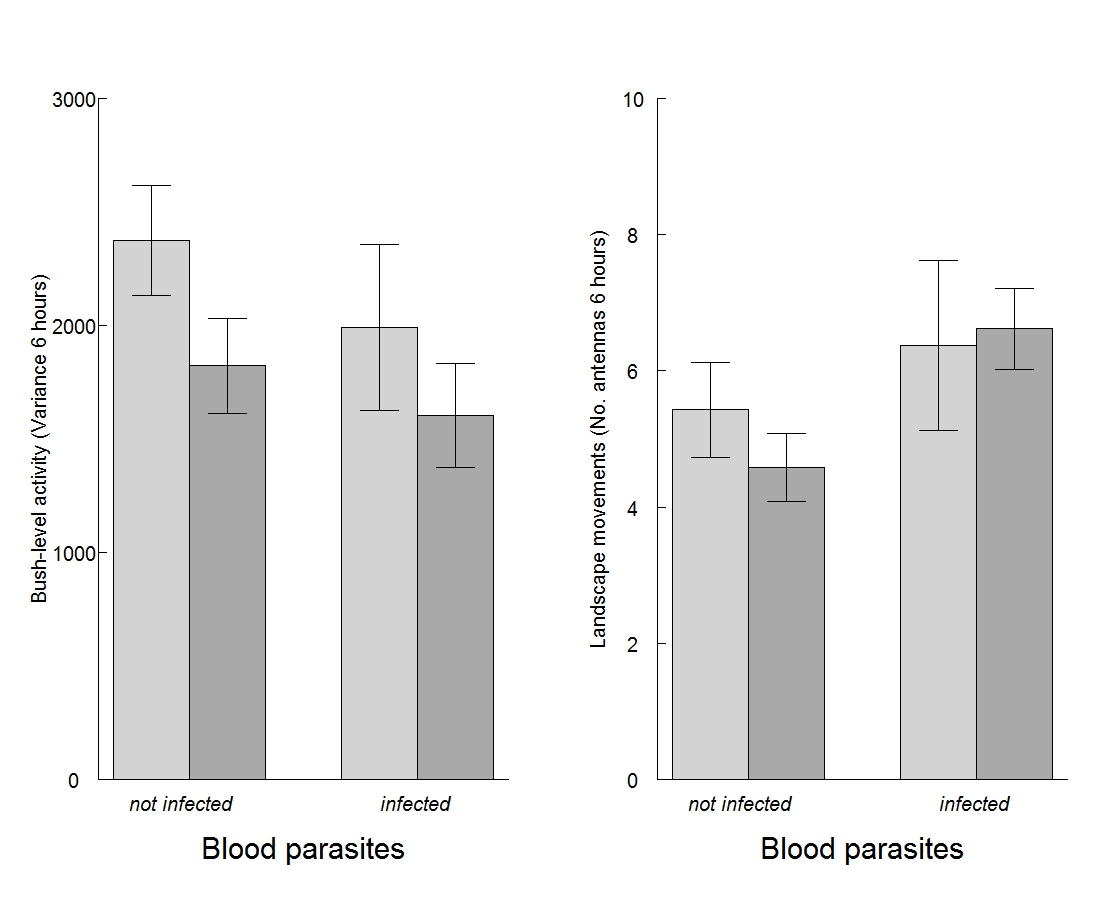


Figure S5: Blood parasite infections in three species of long-distance migrants and three species of short-distance migrants in Falsterbo (Sweden) and their relation to Bush-levels activity patterns and landscape movements.

Table S1: Stopover duration of six songbird species at Falsterbo, Sweden during autumn 2014.

|  |  | Long-distance migrants | | | | | | | | Short-distance migrants | | | | | | | | | | | |
| --- | --- | --- | --- | --- | --- | --- | --- | --- | --- | --- | --- | --- | --- | --- | --- | --- | --- | --- | --- | --- | --- |
| Species | | Common Redstart | | Tree Pipit | | Willow Warbler | | All LDM | | Dunnock | | | | Robin | | | Song Thrush | | | All SDM | |
| Variable |  | Avg | SE | Avg | SE | Avg | SE | Avg | SE | | Avg | SE | Avg | | SE | Avg | | SE | Avg | | SE |
| Stopover duration (days) | C | 1.0 | 0.3 | 1.0 | 0.4 | 1.6 | 0.4 | 1.2 | 0.2 | | 1.3 | 0.4 | 1.0 | | 0.4 | 4.3 | | 1.3 | 2.3 | | 0.6 |
| Lysis titer | C | 2.5 | 0.5 | 3.9 | 0.5 | 0.8 | 0.4 | 2.4 | 0.4 | | 4.1 | 0.3 | 4.4 | | 0.2 | 5.2 | | 0.3 | 4.6 | | 0.2 |
| Agglutination titer | C | 8.6 | 1.2 | 8.3 | 1.1 | 7.6 | 1.5 | 8.1 | 0.7 | | 9.1 | 1.2 | 11.0 | | 0.0 | 8.5 | | 0.9 | 9.5 | | 0.5 |
| Haptoglobin concentration | C | 0.19 | 0.02 | 0.21 | 0.02 | 0.21 | 0.03 | 0.20 | 0.01 | | 0.39 | 0.05 | 0.24 | | 0.01 | 0.43 | | 0.17 | 0.36 | | 0.07 |
| Total Immunoglobulins | C | 51.4 | 4.2 | 61.3 | 1.7 | 45.5 | 2.6 | 54.4 | 2.0 | | 51.3 | 2.4 | 49.9 | | 1.1 | 57.4 | | 2.0 | 53.2 | | 1.2 |
| Bush-level activity (Variance of signal strength) | C | 2023 | 193.7 | 2163 | 574.7 | 2216 | 332.4 | 2141 | 215.8 | | 1587 | 196.4 | 2119 | | 275.6 | 1411 | | 242.0 | 1708 | | 154.8 |
| Landscape movements (no. of antennas) | C | 6.0 | 1.4 | 6.1 | 1.2 | 4.8 | 0.9 | 5.6 | 0.6 | | 6.3 | 1.3 | 4.1 | | 0.1 | 6.6 | | 0.5 | 5.6 | | 0.4 |

Table S2:

Table S3: Capture dates of birds included in this study and for all individuals caught during the standardised ringing scheme by Falsterbo Bird Observatory.

|  |  | Individuals included in this study | | All individuals caught during the standardised ringing scheme (21 Juli - 10 November) | |
| --- | --- | --- | --- | --- | --- |
| species | Migration strategy | Median date | span | Median date | span |
| Tree Pipit | Long-distance migrant | 5/9 | 29/8-10/9 | 27/8 | 14/8-22/10 |
| Common Redstart | Long-distance migrant | 3/9 | 2/9-12/9 | 30/8 | 21/7-14/10 |
| Willow Warbler | Long-distance migrant | 11/9 | 1/9-21/9 | 22/8 | 21/7-28/9 |
| Dunnock | Short-distance migrant | 20/9 | 12/9-29/9 | 20/9 | 22/8-10/11 |
| European Robin | Short-distance migrant | 2/10 | 26/9-11/10 | 30/9 | 22/7-10/11 |
| Song Thrush | Short-distance migrant | 11/10 | 4/10-14/10 | 3/10 | 5/9-10/11 |
